# Paired associative stimulation improves outcomes when applied at the subacute stage after incomplete cervical spinal cord injury

**DOI:** 10.1016/j.neurot.2025.e00778

**Published:** 2025-11-03

**Authors:** Anastasia Shulga, Anna Nätkynmäki, Anna-Lena Pelkonen, Markus Pohjonen, Sarianna Savolainen, Erika Kirveskari, Nina Brandstack, Jyrki P. Mäkelä, Jari Arokoski

**Affiliations:** 1) BioMag Laboratory, HUS Diagnostic Centre, Helsinki University Hospital, University of Helsinki and Aalto University School of Science, Helsinki, Finland; 2) Department of Internal Medicine and Rehabilitation, Division of Rehabilitation, Helsinki University Hospital and University of Helsinki, Helsinki, Finland; 3) Faculty of Sport and Health Sciences, University of Jyväskylä, Jyväskylä, Finland; 4) HUS Medical Imaging Centre, Clinical Neurophysiology, Clinical Neurosciences, Helsinki University Hospital and University of Helsinki, Helsinki, Finland; 5) HUS Medical Imaging Centre, Neuroradiology, Clinical Neurosciences, Helsinki University Hospital and University of Helsinki, Helsinki, Finland

**Keywords:** Paired associative stimulation, Spinal cord injury, Transcranial magnetic stimulation

## Abstract

We conducted a randomized sham-controlled clinical trial from 2019 to 2024 to characterize the safety and efficacy of applying paired-associative stimulation (PAS), consisting of high-intensity transcranial magnetic stimulation and high-frequency peripheral nerve stimulation, at early stages after incomplete spinal cord injury (SCI) to enhance motor recovery. Patients with incomplete cervical SCI were randomized 1:1 within 1–4 months post-injury to receive 12 weeks of PAS or sham stimulation alongside conventional rehabilitation, which was not changed. Patients were followed up to 1.5 years after injury (about 1 year after end of stimulation). Seventeen patients (14 males, age 53 ​± ​16 years) participated. Manual Muscle Test revealed a significant effect of treatment in favor of active group (*F* (1, 470) ​= ​14.69; *p* ​< ​0.001) in muscles that had no antigravity activity before beginning of stimulation. Improvement from baseline was observed at the end of stimulation (active: 346 ​± ​53 ​%, sham: 215 ​± ​26 ​%), 1 year after injury (about 6 months after end of treatment; active: 389 ​± ​61 ​%, sham: 241 ​± ​39 ​%), and at 1.5 years after injury (about 12 months after end of treatment; active: 419 ​± ​73 ​%, sham: 210 ​± ​17 ​%). Greater improvement in fine motor skill tests was observed in the active group. Although the Spinal Cord Independence Measure showed no differences between groups (*p* ​= ​0.36–0.83), there was improvement in activity of daily living tests. The intervention was feasible and well-tolerated in both groups. PAS is a safe and feasible therapy that can be added to conventional rehabilitation even in early stages after SCI.

## Introduction

Spinal cord injury (SCI) severely affects quality of life and imposes a significant economic burden [1]. Worldwide, over 2.5 million people are affected by SCI [2]; a large proportion of SCIs are incomplete, with preserved connectivity [[3], [4], [5]]. Few therapeutic approaches have progressed to clinical practice, and safe, noninvasive, feasible, and timely treatments are needed [[6], [7], [8]]. Strengthening residual pathways after incomplete SCI through non-invasive neuromodulation has gained considerable attention [9,10]. Long-term potentiation (LTP) [11]-like effects, depending on the cooperativity and associativity of neuronal activation, can counteract the connectivity weakness after neuronal trauma and disease. Evidence from animal studies indicates that stimulation inducing spike-time-dependent (STDP)-like plasticity between upper and lower motor neurons is a promising tool for strengthening the residual connectivity and promoting recovery [12,13].

Transient plastic changes in the human corticospinal tract can be induced through paired-associative stimulation (PAS) [[14], [15], [16], [17]]. PAS-induced changes in neuronal connectivity represent a form of STDP [14]. In cortical PAS, transcranial magnetic stimulation (TMS) [18] over the human primary motor or sensory cortex is paired with peripheral electrical nerve stimulation (PNS) of somatosensory afferents to alter neuronal excitability at the cortical level [14,19]. Spinal PAS targets the human spinal cord. In spinal PAS, orthodromic volleys induced by TMS in upper motor neurons and antidromic volleys induced by PNS in lower motor neurons are intended to arrive in a synchronous manner to the corticomotoneuronal synapses of the corticospinal tract [[20], [21], [22], [23]]. Increasing evidence shows that the therapeutic potential of various PAS protocols for incomplete SCI patients is promising and should be further explored [[24], [25], [26], [27], [28]]. More randomized controlled trials are needed to clarify the effect of PAS in patients with SCI [27].

We previously developed a new version of PAS designed as a therapy for incomplete SCI (high-PAS) that utilizes high-intensity TMS and high-frequency PNS. PAS protocols that utilize single-pulse PNS and single-pulse TMS that is slightly above motor threshold utilize the STDP model in which synaptic input to dendrites is active just before a somatic input. However, this model is now considered as simplified [17]. Plasticity induction does not depend only on spike timing but also on firing rate, postsynaptic voltage, and synaptic cooperativity [29]. For example, in experiments using brain tissue slices, connections exhibited classical STDP only when presynaptic and postsynaptic spikes occurred at moderate firing rates (10–20 ​Hz); higher firing rates (>30 ​Hz) induced LTP independent of spike timing [29]. We utilized high-frequency PNS and high-intensity TMS to create multiple interactions at the spinal-cord level and to make the PAS protocol clinically more feasible and the results more stable [30]. TMS delivered at high intensity creates multiple orthodromic volleys [31,32], and high-frequency PNS creates multiple antidromic volleys. Both activations collide at the spinal-cord level [33]. When both LTP and long-term depression (LTD)- producing interactions occur at the same time, LTP wins over LTD [34]. This could explain why the high-PAS protocol produces stable and efficient motor-evoked potential (MEP) amplitude increase in healthy subjects [30,[35], [36], [37], [38], [39]] and stable and long-lasting improvement in motor performance in patients with incomplete SCI [30,[40], [41], [42], [43], [44], [45], [46]]. Due to multiple interactions, the exact site of action is more challenging to define than that for PAS protocols that use single TMS and PNS pulses [30]. However, similar to single-pulse PAS, high-PAS also can specifically target the spinal level, with its outcome depending on the interstimulus interval (ISI) between TMS and PNS [33,47].

Neuromodulation studies requiring long-term administration at subacute stages after neurological insults are challenged by spontaneous recovery and by the overall burden of the acute medical condition. However, it is crucial to investigate whether neuromodulation could safely and non-invasively benefit patients early after injury, since this is the period of greatest plasticity [4] and long-term changes in muscles have not yet occurred [48]. We conducted a double-blind randomized clinical trial to investigate the effect of high-PAS during the early phase after SCI. Treatment was started 1–4 months post injury during inpatient rehabilitation and continued for 12 weeks, including time after discharge home and outpatient rehabilitation. We show that high-PAS modulates motor recovery and particularly promotes restoration of fine movements.

## Methods

The study was registered at clinicaltrials.gov (NCT04101916). The study was approved by the Ethics Committee of Medicine of the Helsinki University Hospital. All patients provided written informed consent.

### Patient selection and randomization

**Fig. 1:**
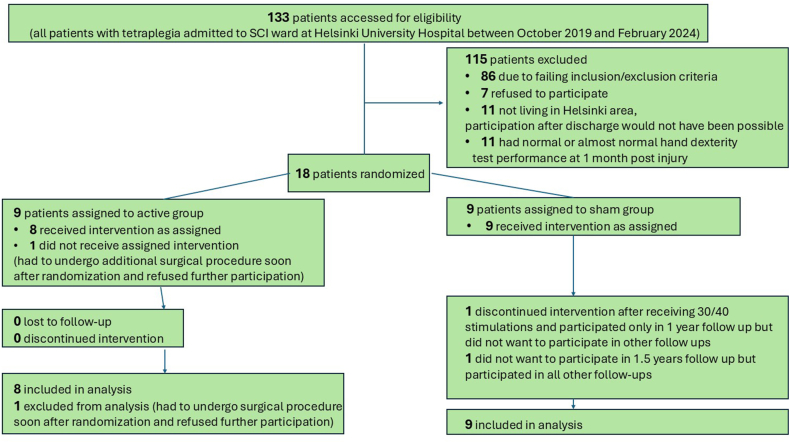
CONSORT flow diagram of the trial.

This trial was double-blind randomized and sham-controlled with a 1:1 allocation ratio. Patients were recruited at the Helsinki University Hospital SCI inpatient ward. Seventeen patients (14 males, mean [±SD] age 53 [±16]) participated (Fig. 1). See Supplementary Methods for details on recruitment, randomization, and blinding.

### Timetable and conventional rehabilitation

The time of stimulations was not linked to physical or occupational therapies. The stimulation schedule minimally affected conventional rehabilitation and patient preferences were considered. Participation did not change the rehabilitation or medications of the patients, and the patient's medical team was not aware of their group allocation.

Stimulations were given 5 times per week during the first 2 weeks and 3 times per week for 10 subsequent weeks. Most stimulations adhered to this schedule, but some small occasional exceptions were allowed if needed (e.g., to accommodate urgent medical situations). Missed sessions were conducted later.

### Stimulation protocol

**Fig. 2:**
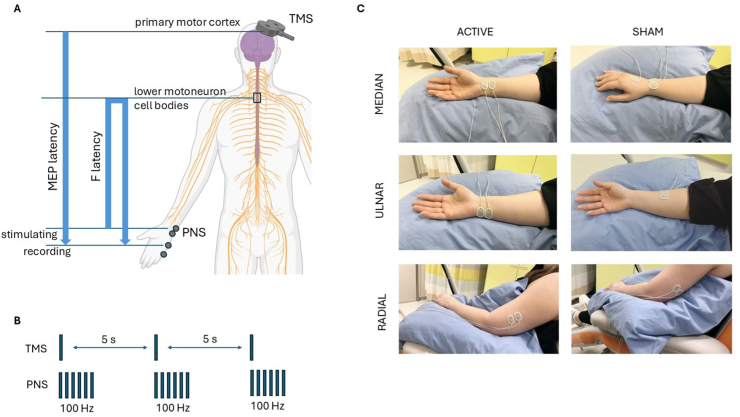
Paired-associative stimulation setup. A, Active setup pre-measurements. Interstimulus interval between TMS and PNS is calculated with the formula [F latency minus MEP latency]. B, Active PNS pattern. C, Position of active and sham PNS electrodes.

See Supplementary Methods and Fig. 2 for a full description of pre-stimulation measurements and stimulation settings. Active stimulation has also been described in detail and discussed previously [30]. Sham stimulation settings were designed to resemble active stimulation as closely as possible.

### Pre-stimulation measurements

Briefly, for active stimulation we measured minimum latencies of F-responses to 0.2-ms pulses at supramaximal intensity from median, ulnar, and radial nerves (Fig. 2) [47]. We also recorded minimum PNS intensity required to produce persistent F-responses to 1-ms pulses for the same nerves to be used later as PNS stimulation intensity. This ensures the use of the lowest possible intensity at which motoneurons of the spinal cord are reached [37,47,49]. Recording electrodes were placed on the abductor pollicis brevis (APB), abductor digiti minimi (ADM), and extensor digitorum (ED) for median, ulnar, and radial nerves, respectively. The same recording electrode placement was used for cortical mapping with TMS as we defined hotspots for APB, ADM, and ED [30]. Fifteen MEPs from each hotspot were sampled and their average latency calculated for ISI. ISI was calculated with the formula F latency – MEP latency] [47]. ISIs varied from −6.8 to +8.7 (see Supplementary data for patient-specific settings).

For sham treatment, stimulation electrodes were not above the motor nerves (Fig. 2) and stimulation settings were trains or three 40-μs pulses at 3 ​Hz. We individually determined minimum intensity to elicit a slight skin sensation that led to individual intensities of 4–15 ​mA. TMS mapping was performed with a 7.5-cm plastic isolator between the coil and head surface. The researcher performing the recordings recreated the active stimulation setup as much as possible. Recording electrodes for TMS responses and PNS were in the same places as in active stimulation.

### Stimulation protocol

**Active:** TMS and PNS were triggered at a pre-defined ISI. TMS was delivered over each hotspot at 100 ​% of stimulator output (SO) paired with PNS of the corresponding nerve (APB with median, ADM with ulnar, and ED with radial). The radial nerve was gently pressed against the skin and the movement elicited by it was monitored to ensure correct activation of the nerve. PNS was delivered (Fig. 2) in trains of six 1-ms pulses at 100 ​Hz [36,39] at intensity determined in pre-measurements [30]. PAS was given every 5 ​s for 20 ​min (240 pairings) for each nerve [36]. If needed, EMLA lidocaine-prilocaine ointment was applied to minimize discomfort from PNS, and PNS intensity was gradually increased to the required level by asking for patient consent for each increase (see Refs. [30,50] for details). TMS was generally tolerable and delivered at 100 ​% SO from the beginning. During stimulation, patients were instructed to very slightly preactivate the muscles innervated by stimulated nerve just before each TMS click (see Supplementary data). If this was not possible or became difficult due to fatigue, the patient was instructed to imagine the corresponding movement [30]. Patients were not allowed to engage in any other activity, such as long discussions or listening to music during stimulation [30,35,51].

**Sham:** Equipment, environment, staff, amount, stimulation duration, and use of motor preactivation or imagery was the same as in the active condition. TMS was delivered at 100 ​% SO with the use of 7.5-cm plastic isolator. PNS was given through electrodes at sham positions (Fig. 2) with trains of three 40-μs pulses at 3 ​Hz at an intensity of just about sensory threshold [52]. Stimulation was triggered every 5 ​s as in active stimulation.

### Outcome measures

Patients were assessed by two physiotherapists with extensive experience in SCI who carefully synchronized their evaluation methods. If possible, each patient had the same physiotherapist at every follow up (12/17 patients).

*Primary endpoints:* Daniels and Worthingham's Manual Muscle Test (MMT) score on a 0–5 scale was sampled from each muscle (see Supplementary Table 1 for test explanation and muscle list) of each hand of each patient before (PRE) and after (POST) treatment, and at 1 year (1Y) and 1.5 years (1.5Y) after injury, corresponding to about 6 months and 12 months after end of treatment, respectively. To avoid the ceiling effect of the test and lack of sensitivity between antigravity (3–5) muscle strength grades [53], muscles with no antigravity activity (0–2) at PRE timepoint, having potential to at least double the score and not requiring evaluation of external resistance, were analyzed separately from muscles having antigravity (3–5) activity at PRE. Spinal Cord Independence Measure (SCIM) was collected from each patient at the same timepoints. Alpha level was adjusted to 0.025 to compensate for two primary endpoints for SCIM, and further to 0.0125 for MMT to compensate for separate analysis of muscles.

*Additional, non-pre-registered outcomes* (exploratory supporting evidence): standard 9-hole peg test (9HPT), Box and Blocks test (BBT), key pinch (KP) and grip strength (GS) tests, activity of daily living (ADL) test custom designed by an experienced SCI physiotherapist (SS, Supplementary Table 2), sensory function (mean average of light touch [LT] and pin prick [PP] scores of International Standards for Neurological Classification of SCI [ISNCSCI] Worksheet), and standard upper and lower extremity spasticity evaluation (Modified Ashworth Scale [MAS]) were sampled from each hand of each patient at the same times as above. One additional test at 6 months after injury (6 ​M) was performed. In addition, patients answered the question 17 of WHOQOL-BREF questionnaire “How satisfied are you with your ability to perform your daily living activities? (over last two weeks)” on a scale of 1–5 (5-very satisfied, 1-very dissatisfied) at the time of MMT and SCIM tests.

### Statistical analysis

Analysis was carried out by original assigned groups. Data are presented as mean ​± ​standard error (SE). For SCIM and “performance in daily life” question, we used Kruskal-Wallis test on IBM SPSS Statistics 30.0. For all other tests, to account for bilateral data, we used linear mixed model on IBM SPSS Statistics 30.0 with treatment and time as fixed effects and a random intercept dependent on the individual included in the model. For MMT, our sample size was sufficiently large to include hand dominance as an additional fixed effect. The model was estimated using the Restricted Maximum Likelihood (REML) method.

All data are presented and analyzed as percent change from each corresponding PRE value ((timepoint value – PRE value)/PRE value∗100). PRE value is the value of the same hand measured within 1 week before beginning treatment. In patients having PRE value zero in one hand (3 patients in 9HPT [2 active ​+ ​1 sham], 2 in BBT [1 ​+ ​1], 2 in ADL [1 ​+ ​1], 2 in KP [1 ​+ ​1], 3 in GS [1 ​+ ​2], and 3 in MAS [1 ​+ ​2]), the PRE value for each hand was the mean average PRE value of both hands to avoid dividing by zero. One patient had zero values for both hands in 9HPT and one in MAS; for these, 0 was substituted by 0.25. For MMT, the PRE value for each muscle is the mean average PRE score of all muscles of the same hand (muscles innervated by stimulated nerves or all measured muscles having 0–2 or 3–4 points at baseline for each corresponding analysis).

## Results

### Primary outcome measures

**Fig. 3:**
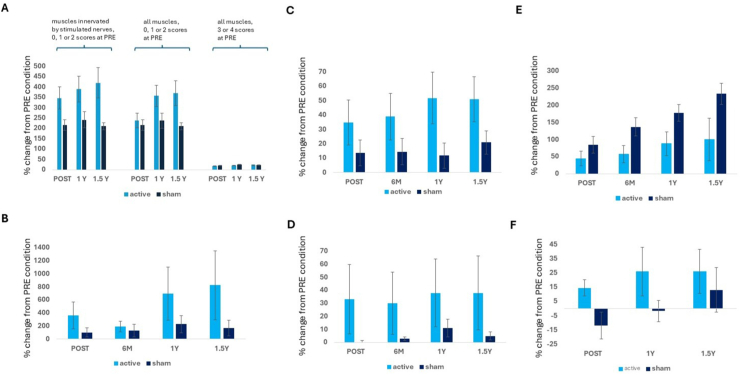
Active group improved more than sham group in MMT, BBT, 9HPT, and ADL tests. 6 ​M, 1Y, and 1.5Y indicate 6 months, 1 year, and 1.5 years since injury, respectively. A, MMT results from muscles with no antigravity activity before stimulation (MMT score 0–2) derived either from muscles innervated only by stimulated median, ulnar, and radial nerves (left) or all measured upper limb muscles (middle) and all measured muscles with antigravity activity (MMT score 3–4) before stimulation. See Supplementary Table 1 for the list of muscles. For muscles with no antigravity activity before stimulation, the improvement in active group was significantly better than in sham group in muscles innervated by stimulated nerves (F (1, 470) ​= ​14.69, *p* ​< ​0.001) and in all muscles (F (1, 543) ​= ​7.28, *p* ​= ​0.007). For muscles with 3–4 antigravity activity before stimulation, sham group performed slightly better (F (1, 2206) ​= ​6.8, *p* ​= ​0.009). B, 9HPT. Active group improved more than sham group (F (1,159) ​= ​4,1, *p* ​= ​0.044). C, BBT. Active group improved more than sham group (F (1, 161) ​= ​10, *p* ​= ​0.002). D, ADL test. Active group improved more than sham group (F (1, 157) ​= ​5.4, *p* ​= ​0.022). E, Grip strength. Active group improved less than sham group (F (1,161) ​= ​4.7, *p* ​= ​0.032). F, Satisfaction with ability to perform daily life activities. There was greater improvement in the active group at POST (*p* ​= ​0.032), but not at 1Y (*p* ​= ​0.27) or 1.5Y(*p* ​= ​0.4).

*MMT:* In muscles innervated by stimulated nerves, we observed a significant effect of time (*F* (1, 470) ​= ​66.66, *p* ​< ​0.001) and treatment in muscles with no antigravity activity (0–2 scores) at PRE (*F* (1, 470) ​= ​14.69, *p* ​< ​0.001). Although both groups improved, the active group had greater percent changes in MMT than the sham group at all time points (Fig. 3A, POST 346 ​± ​53 ​% active vs 215 ​± ​26 ​% sham; 1Y 389 ​± ​61 ​% active *vs*. 241 ​± ​39 ​% sham; 1.5Y 419 ​± ​73 ​% active vs 210 ​± ​17 ​% sham). We observed a significant effect of hand dominance (*F* (1, 470) ​= ​10.85, *p* ​= ​0.001); improvement was stronger in the dominant than non-dominant hand in both groups at all timepoints (350 ​± ​29 ​% dominant vs 197 ​± ​10 ​% non-dominant). When all muscles were analyzed, significant effects of the same direction of time (*F* (1, 543) ​= ​83.79, *p* ​< ​0.001), treatment (*F* (1, 543) ​= ​7.28, *p* ​= ​0.007), and hand (*F* (1, 543) ​= ​11.61, *p* ​< ​0.001) were also found; the size effect and significance of treatment was slightly weaker than in muscles innervated by the stimulated nerves (Fig. 3A, POST 237 ​± ​35 ​% active vs 215 ​± ​26 ​% sham; 1Y 357 ​± ​51 ​% active vs 237 ​± ​36 ​% sham; 1.5Y 369 ​± ​59 ​% active vs 210 ​± ​17 ​% sham). In muscles with antigravity (3–4) activity at timepoint PRE, marginal improvement occurred in both groups (Fig. 3A) and a small difference favored the sham group (*F* (1, 2206) ​= ​6.8, *p* ​= ​0.009) (POST 17 ​± ​1 ​% active vs 20 ​± ​1 ​% sham; 1Y 20 ​± ​1 ​% active vs 25 ​± ​1 ​% sham; 1.5Y 21 ​± ​1 ​% active vs 23 ​± ​1 ​% sham). Muscles with score of 5 ​at PRE were excluded from the analysis as no improvement in these muscles could be expected.

*SCIM:* At the subacute stage, medical staff taught the patients basic selfcare skills reflected by SCIM at different timepoints and pace depending on their overall medical condition, and thus baseline values of SCIM did not reflect pure hand dexterity. No effect of treatment was detected (POST *p* ​= ​0.83, active 24 ​± ​6 ​%, sham 38 ​± ​15 ​%; 1Y *p* ​= ​0.36, active 26 ​± ​6 ​%, sham 55 ​± ​19 ​%; 1.5Y *p* ​= ​0.36, active 27 ​± ​10 ​%, sham 47 ​± ​18 ​%).

### Additional outcomes (exploratory supporting evidence)

*9HPT:* Time (*F* (1, 159) ​= ​6.2, *p* ​= ​0.014) and treatment (*F* (1,159) ​= ​4.1, *p* ​= ​0.044) affected 9HPT performance. Greater improvement was observed in the active than sham group (Fig. 3B).

*BBT:* There was an effect of treatment (*F* (1,161) ​= ​10, *p* ​= ​0.002), with greater improvement in the active than sham group, and of time (*F* (1, 161) ​= ​9.1, *p* ​= ​0.003) (Fig. 3C).

*ADL test:* Treatment affected ADL (*F* (1, 157) ​= ​5.4, *p* ​= ​0.022), which increased more in the active than sham group. Time (*F* (1, 157) ​= ​2.1, *p* ​= ​0.1) did not affect ADL (Fig. 3D). See Fig. 3F for patient satisfaction with ability to perform daily life activities.

*KP:* Time increased pinch strength (*F* (1, 161) ​= ​9.9, *p* ​= ​0.002), with no difference between groups (*p* ​= ​0.61) (POST active 61 ​± ​23 ​%, sham 72 ​± ​29 ​%; 6 ​M active 61 ​± ​23 ​%, sham 77 ​± ​29 ​%; 1Y active 88 ​± ​33 ​%, sham 72 ​± ​29 ​%; 1.5Y active 82 ​± ​41 ​%, sham 128 ​± ​33 ​%).

*GS:* Time (*F* (1, 161) ​= ​23.2, *p* ​< ​0.001) and treatment (*F* (1,161) ​= ​4.7, *p* ​= ​0.032) affected GS. Less improvement was observed in the active than sham group (Fig. 3E).

*Sensory function:* No effect of time (*p* ​= ​0.73) or treatment (*p* ​= ​0.27) was observed (POST active −3±5 ​%, sham 8 ​± ​4 ​%; 6 ​M active 0 ​± ​6 ​%, sham −1 ​± ​4 ​%; 1Y active −3 ​± ​5 ​%, sham 9 ​± ​7 ​%; 1.5Y active 5 ​± ​6 ​%, sham −3 ​± ​3 ​%).

*Spasticity:* Multiple changes occurred in spasticity medication in both groups during the subacute period; 2/6 patients in the active group and 2/8 patients in the sham group had less spasticity medication at 1.5Y than during the stimulation (Table). No effect of time (*p* ​= ​0.38) or treatment (*p* ​= ​0.85) was observed (POST active 14 ​± ​4 ​%, sham 50 ​± ​35 ​%; 6 ​M active −19 ​± ​4 ​%, sham 8 ​± ​27 ​%; 1Y active 0 ​± ​4 ​%, sham 25 ​± ​29 ​%; 1.5Y active 99 ​± ​4 ​%, sham −19 ​± ​22 ​%).

**Table:** Patient characteristics.

Patient number	Etiology of injury	Time since injury at beginning of stimulation, (months ​+ ​days)	Neurological level before stimulation	AIS before stimulation	Right or left-handed	Active or sham	Comorbidities	Conventional rehabilitation, inpatient (times × min/week)	Inpatient weeks out of 12 stimulation weeks	Conventional rehabilitation, outpatient (times × min/week)	CNS-active drugs during stimulation period (at least part of the time) (mg/day)	CNS-active drugs at end of follow-up (mg/day)
1	Cervical spinal stenosis and fall	2 ​+ ​18	C3	D	Right	S	Knee prosthesis, left shoulder operated years before injury	PT 4–5 ​x ​45, OT 4–5 ​x ​60, pool 3 ​× ​60, gym 1–2 ​x ​30	5	PT 1 ​× ​60, OT 1 ​× ​60 or none	Baclofen OD 5–10, mirtazapine OD 3.7–7.5	Baclofen OD 5–20, mirtazapine OD 3.7–7.5
2	Fall	2 ​+ ​10	C1	D	Right	S	Mitral valve prolapse	PT 3–4 ​x ​45, OT 4–5 ​x ​60, gym 2 ​× ​60	5	PT 1 ​× ​45 or none	Tizanidine 6, gabapentin 900, oxycodone OD 5–10, baclofen 5	Tizanidine OD 6
3	Vehicle accident	2 ​+ ​10	C1	D	Right	A	Type II diabetes	PT 4 ​× ​60, OT 4–5 ​x ​60, pool 1 ​× ​60, gym 2 ​× ​45	6	PT 1 ​× ​60 or none, OT 1 ​× ​60	Pregabalin 300-375, mirtazapine 7.5, oxycodone OD 5, baclofen 25, melatonin 5	Baclofen 35, pregabalin 375, melatonin 5
4	Fall	4 ​+ ​11	C2	C	Right	A	Lymphoma years before injury (in remission), history of depression before injury, type II diabetes, hypertension	PT 4–5 ​x ​60, OT 4–5 ​x ​60	12	PT 2 ​× ​60, OT 1 ​× ​60	Escitalopram 10, gabapentin 1200-1800, temazepam OD 10-20	Escitalopram 10, temazepam OD 10-20
5	Fall	1 ​+ ​15	C4	D	Right	S	Migraine	PT 4 ​× ​60, OT 4 ​× ​60	6	PT 2 ​× ​60 or none, OT 1 ​× ​60 or none	Pregabalin 250-300, oxycodone/naloxone 15/7.5, tizanidine OD 2–4, oxycodone OD 10, lorazepam OD 1-2	None
6	Trampoline accident	1 ​+ ​8	C3	D	Left	A	Hypertension, asthma, splenectomy years before injury	PT 4–5x45–60, OT 4–5 ​x ​60, gym 2–3x45	2	PT 0.5–1x60, OT 0.4–1 ​x ​45-60	Pregabalin 300, baclofen 45, oxycodone/naloxone 10, lorazepam 1, oxycodone OD 5–20, amitriptyline 10, zopiclone OD 7.5	Baclofen 60, pregabalin 100, clonazepam OD 0.5
7	Diving accident	2 ​+ ​14	C6	B	Right	S	Asthma	PT 5 ​× ​60, OT 4 ​× ​60, gym 2–3 ​x ​45	8	PT 1.5–2 ​x ​60, OT 1 ​× ​90 or none	Pregabalin 225, quetiapine OD 25	Quetiapine OD 12.5
9	Sledding accident	1 ​+ ​20	C4	D	Left	A	None	PT 4–5 ​x ​60, OT 4–5 ​x ​60	0	PT 0.5 ​× ​60 or none, OT 0.5 ​× ​60 or none	None	None
10	Fall	2 ​+ ​16	C5	D	Right	S	Hypercholesterolemia	PT 4–5 ​x ​60, OT 4–5 ​x ​60, gym 2–3 ​x ​45	7	PT 0.5 ​× ​45, OT 0.5 ​× ​45	Baclofen 15–45, gabapentin 900	Baclofen 45, gabapentin 900
11	Fall	1 ​+ ​29	C5	D	Left	A	Intermittent atrial fibrillation, coronary artery disease, asthma, history of smoking	PT 3–5 ​x ​30–60, OT 3–5 ​x ​30–90, pool 1 ​× ​45, gym 2–3 ​x ​45	9	PT 0.5–1x45–60, OT 0.5 ​× ​60	Baclofen 30–35, pregabalin 375, zopiclone OD 7.5, paracetamol/codeine OD 500/30	Baclofen 5–15, pregabalin 150, paracetamol/codeine OD 500/30
12	Violence	1 ​+ ​24	C4	D	Right	S	None	PT 5 ​× ​45-60, OT 4 ​× ​60, gym 2–3 ​x ​45	9	PT 1–2 ​x ​60 or none, OT 1 ​× ​60 or none	Baclofen 10–50, gabapentin 2100-3600, nortriptyline 25–100, oxycodone-naloxone 15–20, ketamine OD 50–100, oxycodone OD 5–40, temazepam OD 20, buprenorphine 20 ​μg/h, venlafaxine 225	Baclofen 75, gabapentin 3600, oxycodone 40 ​mg x 1, oxycodone OD 5-30
13	Cervical fracture, reason unknown	2 ​+ ​21	C5	B	Right	S	Type II diabetes, hypertension, atrial fibrillation	PT 5 ​× ​60, OT 3–5 ​x ​60–120, gym 2 ​× ​45	12	PT 1 ​× ​60 or none, OT 0.6 ​× ​60, pool 0.5 ​× ​60	Baclofen 30–75, mirtazapine 7.5, pregabalin 300-600, oxycodone OD 5–20, zopiclone OD 7.5, clonazepam 1–1.5, buprenorphine 10 ​μg/h	Baclofen 75, buprenorphine 15 ​μg/h, gabapentin 1800, clonazepam 1.5, tizanidine 4
14	Fall	1 ​+ ​16	C3	D	Right	A	Hip arthroplasty years before injury, diffuse idiopathic skeletal hyperostosis, hypertension, hypercholesterolemia	PT 4–5 ​x ​45–60, OT 3–5 ​x ​60, gym 2 ​× ​45	2	PT 0.5 ​× ​60 or none, OT 0.4 ​× ​60	Baclofen 10, pregabalin 75, temazepam OD 10	None
15	Cervical spinal stenosis	1 ​+ ​18	C1	D	Right	A	Hypertension	PT 4–5 ​x ​45–60, OT 4–5 ​x ​60, gym 2–3 ​x ​60	10	PT 1 ​× ​60, OT 0.7 ​× ​60-90	Gabapentin 1200, baclofen 15	Gabapentin 1200, baclofen 15
16	Spinal infarction	2 ​+ ​7	C3	D	Right	S	None	PT 5 ​× ​45-60, OT 3–5 ​x ​60, pool 1–2 ​x ​45	4	PT 1 ​× ​45, OT 0.7 ​× ​45, pool 0.5 ​× ​45	Gabapentin 600-900, tizanidine OD 2-6	Tizanidine OD 2-6
17	Fall	2 ​+ ​1	C6	D	Right	S	None	PT 3–5 ​x ​30–60, OT 3–5 ​x ​60, gym 2–3 ​x ​45-60	10	PT 1 ​× ​45, OT 0.5 ​× ​45-60, pool 0.5 ​× ​45	Gabapentin 600-1200, baclofen 60	Gabapentin 1500, baclofen 60, tizanidine 8, buprenorphine 5 ​μg/h
18	Fall	3 ​+ ​13	C5	D	Right	A	Hypertension	PT 3–5 ​x ​30–60, OT 3–5 ​x ​60, gym 2–3 ​x ​45-60	7	PT 1.5 ​× ​45, OT 1 ​× ​60, pool 0.5 ​× ​45	Baclofen 25, pregabalin 300-400, melatonin 3	Baclofen 25, melatonin 3

CNS, central nervous system; PT, physiotherapy; OT, occupational therapy; OD, on demand.

*Pain:* A reliable assessment of the effect of the intervention on pain was not feasible due to numerous changes in overall pain medication during the subacute period after SCI and because the complex nature of post-SCI pain is not confined to upper limbs. A total of 5/7 patients in the active and 5/8 patients in the sham group had less neuropathic pain medication at 1.5Y than during the stimulation. For opiate-based medication, the corresponding patient numbers were 2/3 (active) and 3/4 (sham) (Table).

*Possible side effects:* Five patients in the sham group and 4 patients in the active group had occasional overall tiredness or sleepiness during stimulation. One patient was generally more tired during the first 2 weeks of stimulation in the sham group.

In the active group, 1 patient had tension neck and related slight unilateral headache during one of first stimulations, and one patient considered the stimulation-induced hand movements unpleasant. Placing a weight on the hand reduced this sensation.

In the sham group, 1 patient had very mild bilateral tenosynovitis in the wrists, which was resolved with a short etoricoxib treatment. One patient in the sham group felt tiredness in hands at stimulation onset. In the active group, 1 patient had pain in the right wrist during the first four sessions. This affected other rehabilitation, and high-PAS was postponed for 1 month. F-responses were remeasured and stimulation was started with weaker intensity (Supplementary Methods). No pain occurred and stimulation was completed successfully.

No seizures were observed in this study.

## Discussion

Twelve weeks of high-PAS initiated within 1–4 months after SCI improved recovery of weak muscles and improved fine motor control. A stronger recovery and more significant effect were observed in the muscles directly innervated by the stimulated nerves, emphasizing the specificity of the treatment.

Better improvement in MMT of muscles that had no antigravity movement before stimulation in the active group was also associated with better performance in BBT, 9HPT, and ADL test and better satisfaction with daily life activities. This is consistent with our previous results in patients with chronic SCI where high-PAS improved motor performance in upper and lower limbs and was effective for tetra- and paraplegic patients with traumatic and nontraumatic injuries [30,[40], [41], [42], [43], [44], [45], [46]].

GS improved more in the sham than active group and no difference between groups was observed in the KP test. This could be due to compensatory activation patterns and consequent strengthening of less specific neural drive in the sham group supporting mass movements and tenodesis grip, as opposed to improved use of more physiologically correct fine movements enabled by more versatile corticomotor connectivity and improved dexterity in the active group. When applied to SCI patients at the chronic stage, high-PAS did not worsen GS (that had already been developed) and even strengthened grip in some patients [30,[40], [41], [42], [43], [44], [45], [46]].

SCIM was not modified by high-PAS. Participation in the study did not interfere with other rehabilitation. Patients were instructed in selfcare skills reflected by SCIM at different timepoints and pace depending on their overall medical condition. This affected baseline values at the beginning of the study. SCIM does not exclusively reflect hand dexterity, which is the focus of high-PAS treatment, but also strongly depends on cognitive abilities and general health. The custom-made ADL test, which was designed to reflect hand dexterity only, and questions on subjective satisfaction with ADL improved more in the active group. In our previous studies on chronic SCI patients, SCIM improvements were occasionally detected, depending on the length of the stimulation period [30,[40], [41], [42], [43], [44], [45], [46]].

Sensory functions or spasticity were not modified by high-PAS, consistent with previous results from chronic SCI patients [30,[40], [41], [42], [43], [44], [45], [46]]. As spasticity and pain medication change rapidly during the subacute period, interpretation of the possible modifications is challenging. Reduction of spasticity and pain medication over time was as evident in the active as in the sham group, suggesting that active treatment did not increase pain or spasticity. We are aware that drugs can modify the efficacy of neuroplasticity-inducing treatments [54,55], but evaluation of drug effects on outcomes was not possible in this study. The use of standard-of-care drugs (Table) did not prevent the high-PAS effect.

Sessions were incorporated into inpatient and outpatient rehabilitation. Although patients perceived this addition as somewhat time-consuming, they were motivated to spend extra hours for an additional therapeutic opportunity. As opposed to other paired stimulation protocols that require combination of exercise simultaneously or immediately after the stimulation [[56], [57], [58]], high-PAS does not require synchronization with training, making its incorporation into rehabilitation schedules relatively easy. Although the high-PAS effect is specific for ISI [33], small deviations in calculating ISI are inevitable in clinical practice where neurophysiological recordings can be compromised (e.g., by spasticity, which does not prevent effective MEP increase by high-PAS) [38]. Single high-PAS sessions do not induce sympathetic nervous system activation in healthy subjects [59]. High-PAS thus appears both safe and feasible as an addition to conventional SCI rehabilitation.

The effects of the stimulation were not limited to the muscles innervated by the stimulated nerves, although the effect in muscles belonging to these nerves was more profound, highlighting the specificity of the stimulation. In high-PAS, the PNS train is applied with an intensity that is sufficient to activate lower motoneuron cell bodies at the spinal-cord level, as ensured by F-response measurements [37]. Therefore, a PNS-induced pulse train applied to a peripheral branch is thought to induce activation of the larger pool of the lower motoneurons and intervening interneurons and together with TMS promote plastic changes at the spinal-cord level between upper and lower motoneurons and their interneurons [30]. In our previous studies where measurements for high-PAS were made utilizing MEP and F-responses obtained from electrodes placed on the abductor hallucis and stimulation targeting the tibial nerve, changes in H-reflex pathway measured from the soleus confirmed the hypothesis that changes occur at the spinal level affecting the targeted myotome [33]. Activation of the surrounding regions of the motor cortex by high-intensity TMS is also very plausible [30].

This study had limitations. As expected for the subacute period, fluctuations in the patients’ overall medical condition, drug dosages, and other factors were evident within both groups, and performance expectedly increased in both groups. Baseline values were affected by the exact time of the treatment start, injury severity, and other patient-specific factors. Moreover, the overall number of participants was relatively small. Despite this, we detected a significant effect of high-PAS. Although patients with psychiatric comorbidities, progressive conditions, or age <18 or >75 years were excluded, in clinical practice high-PAS could also be effective for such patients. Further studies are also needed to characterize the role of factors such as extent of lower motor neuron damage that affect responsiveness to high-PAS [40,60,61].

No seizures were observed in this study. 1-Hz stimulation is considered low frequency [18]; here we used 0.2 ​Hz with single pulses. For low frequencies, seizure induction is considered rare, or this type of stimulation can be considered even protective [18]. Frequencies <1 ​Hz have been used without incident and no recommendations exist on maximal intensities for such frequencies in safety guidelines [62]; this has not been changed in later updates [18,63]. PAS protocols in general have not been associated with seizures [63]. Here, brain pathologies have been excluded prior to participation by brain MRI evaluated by a neuroradiologist. If including patients with brain pathologies in clinical work, safety guidelines should be consulted concerning allowed stimulation settings and management of possible related adverse events [18,62,63].

In conclusion, this study is the first sham-controlled clinical trial to demonstrate that high-PAS is a safe, feasible, and potentially effective addition to rehabilitation after incomplete SCI even at a very early stage after injury. The subacute phase was selected for high-PAS intervention because neuroplasticity is believed to peak early after SCI, offering a critical window for recovery. At this stage, weaker descending pathways still retain better partial function, and muscles have not yet undergone irreversible degeneration. Our findings suggest that applying stimulation during this period can effectively engage residual neural circuits and promote motor improvement. The use of high-PAS for other patient groups also warrants further investigation. High-PAS could also be an attractive option as an additional therapy combined with experimental stem cells and pharmacological treatments that must be administered at very early stages after SCI [2,7].

## Author contributions

Conception or design of the work: AS, A-LP, SS, EK, JM, JA. Acquisition, analysis or interpretation of data: AS, AN, A-LP, MP, SS, EK, NB. AS drafted the manuscript. All authors reviewed it critically for important intellectual content, approved final version and agree to be accountable for all aspects of the work in ensuring that questions related to the accuracy or integrity of any part of the work are appropriately investigated and resolved. AS had full access to all the data in the study and takes responsibility for the integrity of the data and the accuracy of the data analysis.

## Data availability

Data is available at Fairdata storage service.

https://doi.org/10.23729/fd-0cfe8125-c95e-307b-9120-779ce3a8f9c5.

## Declaration of competing interests

The authors declare the following financial interests/personal relationships which may be considered as potential competing interests: Anastasia Shulga reports financial support was provided by Wings for Life Spinal Cord Research Foundation. Anastasia Shulga reports financial support was provided by Research Council of Finland. Other authors declare that they have no known competing financial interests or personal relationships that could have appeared to influence the work reported in this paper.
