# Supplementary material for: Paired associative stimulation improves outcomes when applied at the subacute stage after incomplete cervical spinal cord injury
Source: Neurotherapeutics. 2025 Nov 3;23(1):e00778. doi: 10.1016/j.neurot.2025.e00778 (PMC12976545; doi:10.1016/j.neurot.2025.e00778)
Supplement: Multimedia component 1 [file mmc1.docx]

**Supplement**

Supplementary Table 1 MMT scale and list of measured muscles (page 2)

Supplementary Table 2 ADL test (page 4)

Supplementary Methods (page 5)

**eTable 1**

**MMT scale and list of measured muscles**

**Grades**

**0**: No visible or palpable contraction

**1**: Visible or palpable contraction without motion

**2**: Full range of motion, gravity eliminated

**3**: Full range of motion against gravity

**4:** Full range of motion against gravity, moderate resistance

**5**: Full range of motion against gravity, maximal resistance

**List of measured muscles and innervation** (stimulated muscles are innervated by median, ulnar, or radial nerves)

| MUSCLE | INNERVATION (MEDIAN, ULNAR, RADIAL OR OTHER) |
| --- | --- |
| serratus anterior | other |
| trapezius pars cran., levator scapulae | other |
| trapezius pars med. | other |
| rhomboid minor & major | other |
| deltoideus pars vent. | other |
| deltoideus post, latissimus dorsi, teres major | other |
| deltoideus pars lat, supraspinatus | other |
| deltoideus pars post. | other |
| pectoralis major | other |
| infraspinatus, teres minor | other |
| subscapularis, pectoralis major, latissimus dorsi, teres major | other |
| biceps brachii, brachialis, brachioradialis | other |
| triceps brachii | RAD and other |
| supinator longus, biceps brachii | RAD and other |
| pronator teres, pronator quadratus | MED |
| MUSCLE | INNERVATION (MEDIAN, ULNAR, RADIAL OR OTHER) |
| flexor carpi radialis | MED |
| flexor carpi ulnaris | ULN |
| extensor carpi radialis | RAD |
| extensor carpi ulnaris | RAD |
| PIP II-V flexor digitorum superficialis | MED |
| DIP II-III flexor digitorum profundus I-II | MED |
| DIP IV-V flexor digitorum profundus IV-V | ULN |
| MP II-V extensor digitorum | RAD |
| extensor digiti minimi | RAD |
| extensor indicis | RAD |
| abduction II-V interossei dorsalis | ULN |
| abductor digiti minimi | ULN |
| adduction II-V interossei palmares | ULN |
| MP flexor pollicis brevis | MED-ULN |
| IP flexor pollicis longus | MED |
| MP extensor pollicis brevis | RAD |
| IP extensor pollicis longus | RAD |
| abductor pollicis brevis | MED |
| abductor pollicis longus | RAD |
| adductor pollicis | ULN |
| opponens pollicis | MED |
| V finger opponens digiti minimi | ULN |

**eTable 2**

**Activities of Daily Living (ADL) test**

Each hand is rated separately for each task and given a score of 0 (not capable of performing task) or 1 (capable of performing task). The test is performed by a physiotherapist. Buttoning and unbuttoning task is rated for both hands (0, not capable; 2, capable). Maximum test score for both hands is 20.

| Using spoon |
| --- |
| Cutting bread |
| Opening a bottle with a screw cap |
| Pouring water from a bottle to a glass |
| Drinking water from a glass |
| Taking an object from diagonal upfront |
| Buttoning and unbuttoning (3 buttons) |
| Pencil grip and drawing a triangle |
| Using a pad/tablet and writing a name on it |
| Opening a lock (bathroom door) |

**Supplementary Methods**

**Patient recruitment**

Inclusion criteria were spinal cord injury (SCI) caused by external trauma or SCI caused by nontraumatic non-progressive disease of the spinal cord, cervical level injury (tetraplegia), time from injury/onset of symptoms 1-4 months, medical condition stable, and present residual neural connectivity in upper extremity (some voluntary activity in finger muscles or motor-evoked potentials [MEP] obtainable from distal hand muscles).

Exclusion criteria were diagnosed brain damage (visible in magnetic resonance imaging [MRI] or computer tomography [CT]), no activity in hands/fingers and no MEPs recorded from distal hand muscles, epilepsy, metal inclusion in the head area, high intracranial pressure, pacemaker, implanted hearing device, progressive diseases of spinal cord or brain (e.g. malignant tumours, degenerative diseases), history of malignant tumour within the past 5 years, previous head or SCI affecting the motor performance of upper extremities, congenital anomaly in the anatomical structure of spinal canal/cord or dura, significant systemic disease or other condition that could cause neurological deficit or may affect subject’s ability to undergo investigation-related procedures, acute severe infection, contraindications for MRI, current severe psychiatric diseases, current chronic drug and/or alcohol abuse, pregnancy, severe emergency care polyneuropathy, and pressure ulcer affecting the subject’s ability to undergo the procedure safely. We excluded 11 patients in total (Figure 1) due to normal or almost normal performance in hand dexterity tests at 1 month post injury.

Male and female patients aged 18-75 years with cervical SCI were eligible for the study. The initial eligibility age range was 18-70 years. Two months after the start of the trial this was changed to 18-75 years, as the COVID-19 pandemic limited the availability of patients. Moreover, patients aged 70-75 years were considered as potentially suitable for participation if other inclusion criteria were met and exclusion criteria did not prevent participation.

Approximately 30 tetraplegic patients are rehabilitated at the ward yearly. We expected that approximately 7 patients per year would be eligible. We aimed at 20 patients from years 2020-2022, but due to the COVID-19 pandemic at the beginning of the trial, recruitment was slower than expected and the trial was terminated when 18 patients were recruited and resources allocated for the trial were used.

**Randomization and blinding**

Randomization was performed by a biostatistician consultant at Helsinki University Hospital for 20 patients with block randomization with variable block sizes using the blockrand-package [1]. The allocation sequence was sent by the biostatistician directly to the research assistant (A-LP), who made non-transparent envelopes with patient numbers and allocation. AS and MP screened and recruited patients based on eligibility and inclusion and exclusion criteria. The final recruitment decision was made by AS. AS and MP did not have access to the allocation sequence. To assess the exclusion criteria “Diagnosed brain damage, visible in MRI or CT”, patients otherwise eligible for and interested in participation underwent brain MRI assessed by a neuroradiologist. The allocation envelope was opened after the patient was recruited and signed informed consent was acquired. At this point, the research assistant (AN or A-LP) and AS were no longer blinded to treatment as this was not possible regarding practical implementation or patient safety; AS, AN, and A-LP did not participate in patient evaluation. Patients, two physiotherapists who assessed patient outcomes, and ward personnel caring for the patients were blinded to treatment. Patients were informed of their allocation after the last follow up.

**Integrity of the blind**

After the last stimulation, patients were asked which group they believed to belong to. In the active group of 8 patients, 5 patients believed they were in active group, 1 believed they were in sham group, and 2 said they could not guess. In the sham group of 9 patients, 2 believed they were in active group, 2 believed they were in sham group, and 5 said they could not guess.

Physiotherapists who assessed patients were asked if they had an opinion of which group the patient most probably belonged to after the last follow-up measurement. Out of 17 patients, for 13 patients they said they could not guess and for 4 patients they had an opinion (3 were incorrect and 1 was correct).

**Pre-stimulation measurements**

We used a Keypoint device (Natus Medical Inc., Pleasanton, CA, USA) for F-response measurement and PNS and an eXimia magnetic stimulator (Nexstim Ltd., Helsinki, Finland) with cooled figure-of-eight coil (outer diameter 70 mm) for TMS. Self-adhering surface electrodes were Neuroline 720, AMBU A/S, Ballerup, Denmark.

**Active:** Active treatment pre-stimulation measurements were performed as described previously [2]. We measured minimum latencies of F-responses from 10 responses to 0.2-ms pulses at supramaximal intensity from median, ulnar, and radial nerves to be used later for calculation of interstimulus interval (ISI) between transcranial magnetic stimulation (TMS) and peripheral electrical nerve stimulation (PNS) [3]. We also recorded the minimum PNS intensity required to produce persistent F-responses to 1-ms pulses for the same nerves to determine PNS stimulation intensity for each nerve. This ensured use of the lowest possible intensity at which motoneurons of the spinal cord are reached [3–5]. Stimulating electrode positions are shown in Figure 2 in the main document (see also [2,6]). Recording electrodes were placed on abductor pollicis brevis (APB), abductor digiti minimi (ADM), and extensor digitorum (ED) for median, ulnar, and radial nerves, respectively. The same recording electrode placement was used for cortical mapping with TMS as we defined hotspots (motor cortex sites where largest and most consistent MEPs were elicited) for APB, ADM and ED (see Shulga et al [2] for more details). Fifteen MEPs from each hotspot were sampled and their average latency calculated for ISI. ISI was calculated with the formula F latency – MEP latency [3]. See tables below for individual stimulation settings.

**PNS intensities, active group, mA:**

| patient number | right median | right ulnar | right radial | left median | left ulnar | left radial |
| --- | --- | --- | --- | --- | --- | --- |
| 3 | 15.7 | 13.5 | 15 | 15.8 | 13 | 19 |
| 4 | 13 | 13* | 11 | 5 | 12 | 9 |
| 6 | 4.5-12.5** | 7-11** | 8 | NS | NS | NS |
| 9 | NS | NS | NS | 8 | 5 | 9 |
| 11 | 4 | 15 | 4.5 | 4 | 5 | 4 |
| 14 | 6.5 | 7.5 | 18 | 4.5 | 10 | 24 |
| 15 | 4.5 | 2 | 13 | 4 | 2.5 | 6.5 |
| 18 | 3 | 8 | 22 | 2 | 3 | 11 |

NS, not stimulated.

* response was not found; we used same intensity as for right median nerve

** at first measurement, we obtained values 30 (median), 11 (ulnar), and 8 (radial). The patient’s wrist became painful, so we took a 1-month pause and remeasured median and ulnar settings with preactivation, which lowered the threshold to 4.5 (median) and 7 (ulnar). After this, stimulations were successfully performed at these intensities. Intensities were gradually increased to 12.5 (median) and 11 (ulnar) towards the last stimulation sessions.

**F latencies, active group, ms**

| patient number | right median | right ulnar | right radial | left median | left ulnar | left radial |
| --- | --- | --- | --- | --- | --- | --- |
| 3 | 30.0 | 29.6 | 25.0 | 29.4 | 29.8 | 23.0 |
| 4 | 31.0 | NM | 21.4 | 33.6 | 35.8 | 20.1 |
| 6 | 32.0 | 32.0 | 20.0 | NS | NS | NS |
| 9 | NS | NS | NS | 28.3 | 30.6 | 12.7 |
| 11 | 30.3 | 27.4 | 13.0 | 32.0 | 30.2 | 15.0 |
| 14 | 29.8 | 29.8 | 22.8 | 29.1 | 29.8 | 20.0 |
| 15 | 32.7 | 29.8 | 25.8 | 31.8 | 33.0 | 24.8 |
| 18 | 27.4 | 27.4 | 18.0 | 26.7 | 27.3 | 23.0 |

NM, not measurable; NS, not stimulated.

**MEP latencies, active group, ms**

| patient number | right ABP | right ADM | right ED | left APB | left ADM | left ED |
| --- | --- | --- | --- | --- | --- | --- |
| 3 | 25.8 | 28.6 | 18.0 | 23.5 | 26.0 | 20.1 |
| 4 | 27.8 | 29.1 | 14.1 | 29.1 | 27.1 | 15.0 |
| 6 | 33.4 | 26.7 | 18.2 | NS | NS | NS |
| 9 | NS | NS | NS | 23.9 | 21.9 | 14.6 |
| 11 | 26.7 | 25.8 | 17.8 | 36.5 | 29.2 | 16.3 |
| 14 | 29.6 | 38.2 | 15.1 | 27.9 | 36.6 | 14.5 |
| 15 | 31.4 | 35.4 | 18.2 | 33.8 | 28.3 | 17.5 |
| 18 | 24.6 | 24.5 | 14.5 | 22.9 | 24.9 | 14.4 |

ABP, abductor pollicis brevis; ADM, abductor digiti minimi; ED, extensor digitorum; NS, not stimulated.

**ISI latencies, active group, ms** (calculated from above values with F-MEP formula [3])

| patient number | right ABP | right ADM | right ED | left APB | left ADM | left ED |
| --- | --- | --- | --- | --- | --- | --- |
| 3 | 4.2 | 1 | 7 | 5.9 | 3.8 | 2.9 |
| 4 | 3.2 |  | 7.3 | 4.5 | 8.7 | 5.1 |
| 6 | -1.4 | 5.3 | 1.8 | NS | NS | NS |
| 9 | NS | NS | NS | 4.4 | 8.7 | -1.9 |
| 11 | 3.6 | 1.6 | -4.8 | -4.5 | 1 | -1.3 |
| 14 | 0.2 | -8.4 | 7.7 | 1.2 | -6.8 | 5.5 |
| 15 | 1.3 | -5.6 | 7.6 | -2 | 4.7 | 7.3 |
| 18 | 2.8 | 2.9 | 3.5 | 3.8 | 2.4 | 8.6 |

ABP, abductor pollicis brevis; ADM, abductor digiti minimi; ED, extensor digitorum; NS, not stimulated.

Negative value indicates that TMS preceded PNS; and positive value indicates that PNS preceded TMS.

**Resting motor thresholds (RMT), active group, % of stimulator output** (not used for stimulation settings). Preactivation or motor imagery were used for mapping if RMT was over 100.

| patient number | right ABP | right ADM | right ED | left APB | left ADM | left ED |
| --- | --- | --- | --- | --- | --- | --- |
| 3 | 35 | 36 | 34 | 38 | 41 | 33 |
| 4 | >100 | >100 | >100 | >100 | >100 | >100 |
| 6 | 98 | >100 | 56 | NS | NS | NS |
| 9 | NS | NS | NS | 38 | 33 | 31 |
| 11 | 70 | 80 | 44 | >100 | 60 | 35 |
| 14 | 52 | 55 | 35 | 49 | 67 | 37 |
| 15 | 66 | 76 | 39 | 40 | 54 | 47 |
| 18 | >100 | 64 | 50 | 54 | 70 | 40 |

NS, not stimulated.

**Sham:** F-response measurement was performed seemingly as in active group, but stimulation electrodes were not above the motor nerves (Figure 2 in the main document) and stimulation settings were the same as those used later for sham stimulation (trains of three 40-µs pulses at 3 Hz). We individually determined minimum intensity to elicit slight skin sensation with this electrode position and settings. Individual intensities were between 4 mA and 15 mA, depending on the sensory threshold. Stimulation intensity was not further increased if threshold was >15 mA. TMS mapping was performed in the same way as in active condition but with a 7.5-cm plastic isolator between the coil and head surface. Real MEPs and F-responses were not elicited and thus could not be recorded, but the researcher performing both recordings acted as close to the active stimulation setup as possible. Recording electrodes for TMS responses and PNS were in the same places as in active stimulation.

**Stimulation protocol**

**Active:** TMS and PNS were triggered with Presentation® software (Neurobehavioral System Inc., Albany, NY, USA) at pre-defined ISI calculated as described above (see tables above for individual settings). TMS was delivered over each hotspot at 100% of stimulator output (SO) paired with PNS of corresponding nerve (ABP with median, ADM with ulnar, and ED with radial). The radial nerve was gently pressed against the skin and the movement elicited by it monitored to ensure correct activation of the nerve. PNS was delivered through stimulating electrodes (Figure 2 in the main document) in trains of six 1-ms pulses at 100 Hz [7,8] at intensity determined in pre-measurements [2]. PAS was given every 5 s for 20 min (240 pairings) for each nerve [8]. Stimulation of 6 nerves (both hands) thus took 2 hours in total plus general preparation time (30-40 minutes). One hand of patients 6 and 9 (active) and 5 and 16 (sham) had less than 2 muscles below grade 5 and these hands were not stimulated; for these patients, stimulation time was 1 hour plus approximately 30 min for preparations. If needed, EMLA lidocaine-prilocaine ointment was applied to minimize discomfort from PNS, and PNS intensity was ramped up gradually to the required level after asking for patient consent for each increase (see [2,9] for details). TMS was generally tolerable and delivered at 100% SO from the beginning. Motor cortex-nerve pairs were mostly stimulated in randomized order; however, some exceptions were allowed for practical reasons, such as EMLA use. During stimulation, patients were instructed to very slightly preactivate the muscles innervated by the stimulated nerve just before each TMS click (see list of the movements below). If this was not possible or became difficult due to fatigue, the patients were instructed to imagine the corresponding movement [2]. Patients were not allowed to engage in any other activity, such as long discussions or listening to music during stimulation, as this could affect the outcome [2,10,11]. In some sessions, patients 4 and 18 had difficulties staying awake and listening to music was allowed [11].

**Sham:** Equipment, environment, staff, amount and duration of stimulations, and use of motor preactivation or imagery was the same as in the active condition. TMS was delivered at 100% SO to obtain the same clicking sound as in active stimulation with the use of a 7.5-cm plastic isolator. PNS was given through electrodes at sham positions (Figure 2 in the main document) with trains of three 40-µs pulses at 3 Hz at an intensity just above sensory threshold (individually determined at pre-stimulation measurements) to ensure that the stimulation created a sensation but did not reach motor nerves [12]. Stimulation was triggered every 5 s as in active stimulation.

**Preactivation movements (both groups)**

Median nerve/APB: opposition of I-II-III fingers

Ulnar nerve/ADM: spreading fingers (half of the time), flexion of IV-V fingers (half of the time)

Radial nerve/ED: wrist and finger extension

Patients were instructed to perform very slight movements as described above just before each TMS click. If movement was not possible, they were instructed to imagine it.

References

[1] Snow, G. blockrand: Randomization for block random clinical trials. R package version 2013.

[2] Shulga A, Lioumis P, Kirveskari E, Savolainen S, Makela JP. A novel paired associative stimulation protocol with a high-frequency peripheral component: a review on results in spinal cord injury rehabilitation. The European Journal of Neuroscience 2021. https://doi.org/10.1111/ejn.15191 [doi].

[3] Shulga A, Lioumis P, Kirveskari E, Savolainen S, Makela JP, Ylinen A. The use of F-response in defining interstimulus intervals appropriate for LTP-like plasticity induction in lower limb spinal paired associative stimulation. Journal of Neuroscience Methods 2015;242C:112–7. https://doi.org/S0165-0270(15)00013-8 [pii].

[4] Mesrati F, Vecchierini MF. F-waves: neurophysiology and clinical value. Neurophysiologie Clinique = Clinical Neurophysiology 2004;34:217–43. https://doi.org/10.1016/j.neucli.2004.09.005.

[5] Pohjonen M, Nyman A-L, Kirveskari E, Arokoski J, Shulga A. Optimal peripheral nerve stimulation intensity for paired associative stimulation with high-frequency peripheral component in healthy subjects. Sci Rep 2022;12:12466. https://doi.org/10.1038/s41598-022-16811-1.

[6] Shulga A, Lioumis P, Zubareva A, Brandstack N, Kuusela L, Kirveskari E, et al. Long-term paired associative stimulation can restore voluntary control over paralyzed muscles in incomplete chronic spinal cord injury patients. Spinal Cord Series and Cases 2016;2:16016. https://doi.org/10.1038/scsandc.2016.16 [doi].

[7] Tolmacheva A, Makela JP, Shulga A. Increasing the frequency of peripheral component in paired associative stimulation strengthens its efficacy. Scientific Reports 2019;9:3849-019-40474–0. https://doi.org/10.1038/s41598-019-40474-0 [doi].

[8] Mezes M, Havu R, Tolmacheva A, Lioumis P, Makela JP, Shulga A. The impact of TMS and PNS frequencies on MEP potentiation in PAS with high-frequency peripheral component. PloS One 2020;15:e0233999. https://doi.org/10.1371/journal.pone.0233999 [doi].

[9] Gajraj NM, Pennant JH, Watcha MF. Eutectic mixture of local anesthetics (EMLA) cream. Anesthesia and Analgesia 1994;78:574–83.

[10] Stefan K, Wycislo M, Classen J. Modulation of associative human motor cortical plasticity by attention. Journal of Neurophysiology 2004;92:66–72. https://doi.org/10.1152/jn.00383.2003 [doi].

[11] Holopainen K, Sihvonen AJ, Kauramäki J, Särkämö T, Shulga A. The effects of music combined to paired associative stimulation on motor-evoked potentials and alertness in spinal cord injury patients and healthy subjects. Sci Rep 2024;14:10194. https://doi.org/10.1038/s41598-024-60984-w.

[12] Zundert, A, Hadzic, A. Electrical Nerve Stimulators and Localization of Peripheral Nerves n.d.
